# Salivary complement inhibitors from mosquitoes: Structure and mechanism of action

**DOI:** 10.1074/jbc.RA120.015230

**Published:** 2020-11-24

**Authors:** Ethan C. Strayer, Stephen Lu, Jose Ribeiro, John F. Andersen

**Affiliations:** Laboratory of Malaria and Vector Research, NIH-NIAID, Rockville, Maryland, USA

**Keywords:** blood feeding, C3bBb, properdin, X-ray crystallography, aHUS, atypical hemolytic uremic syndrome, AMD, age-related macular degeneration, AP, alternative pathway, CP, classical pathway, LP, lectin pathway, NHS, normal human serum, PDS, properdin-depleted serum, PNH, paroxysmal nocturnal hemoglobinuria, SPR, surface plasmon resonance

## Abstract

Inhibition of the alternative pathway (AP) of complement by saliva from *Anopheles* mosquitoes facilitates feeding by blocking production of the anaphylatoxins C3a and C5a, which activate mast cells leading to plasma extravasation, pain, and itching. We have previously shown that albicin, a member of the SG7 protein family from *An. Albimanus*, blocks the AP by binding to and inhibiting the function of the C3 convertase, C3bBb. Here we show that SG7.AF, the albicin homolog from *An. freeborni*, has a similar potency to albicin but is more active in the presence of properdin, a plasma protein that acts to stabilize C3bBb. Conversely, albicin is highly active in the absence or presence of properdin. Albicin and SG7.AF stabilize the C3bBb complex in a form that accumulates on surface plasmon resonance (SPR) surfaces coated with properdin, but SG7.AF binds with lower affinity than albicin. Albicin induces oligomerization of the complex in solution, suggesting that it is oligomerization that leads to stabilization on SPR surfaces. Anophensin, the albicin ortholog from *An. stephensi*, is only weakly active as an inhibitor of the AP, suggesting that the SG7 family may play a different functional role in this species and other species of the subgenus *Cellia*, containing the major malaria vectors in Africa and Asia. Crystal structures of albicin and SG7.AF reveal a novel four-helix bundle arrangement that is stabilized by an N-terminal hydrogen bonding network. These structures provide insight into the SG7 family and related mosquito salivary proteins including the platelet-inhibitory 30 kDa family.

Feeding by mosquitoes and other hematophagous arthropods elicits host responses aimed at preventing blood loss and controlling microbial infection (1). Ultimately, these host responses can affect the transmission of parasites and viruses by limiting feeding success. Pertinent host responses to feeding include activation of the hemostatic system (coagulation cascade, vasoconstrictive mechanisms, and platelet activation) as well as immediate inflammatory/antimicrobial responses (activation of mast cells and the complement system). Salivary antihemostatic factors have been well characterized and include inhibitors of coagulation proteases, apyrases, disintegrins, and scavengers of procoagulant small molecules (2, 3, 4, 5). Known anti-inflammatory mediators consist mainly of binders of biogenic amines and leukotrienes, peptides, and protease inhibitors (6, 7, 8, 9). Inhibitors of the complement system have also been identified from mosquitoes, ticks, sand flies, and triatomine bugs suggesting that activated complement in the blood meal is deleterious to feeding and the digestion of blood (10, 11, 12, 13, 14, 15, 16). The complement system is a proteolytic cascade that is rapidly induced by microbial infection or tissue damage leading to opsonization of microbes, synthesis of microbicidal membrane attack complexes, and production of proinflammatory anaphylatoxins. These processes lead to the phagocytosis of invading pathogens, lysis of pathogen membranes, and induction of antimicrobial inflammatory responses. Arthropod-produced complement inhibitors attack various points in the classical (CP), lectin (LP), alternative (AP), and common pathways of the complement cascade. These pathways of complement activation are initiated differently but result in the conversion of the plasma protein C3 to its activated form, C3b, by proteolytic convertase complexes (17). Newly formed C3b reacts with nucleophilic groups by means of its labile thioester moiety and becomes covalently linked at the microbial surface. Once attached, it binds with a serine protease zymogen, factor B, which is then cleaved by a second serine protease, factor D to form a complex known as the alternative C3 convertase, C3bBb, which itself cleaves C3 to form C3b. At this point, the complement response is enormously amplified as each C3bBb complex produces many C3b molecules, each with the potential of binding to a microbial surface and forming a new alternative convertase complex. Downstream steps of the common pathway of complement include the activation of C5, a key component of the membrane attack complex, by the C5 convertase, which also requires C3b and thus depends on amplification of the alternative convertase (17). The plasma protein properdin is essential for normal C3bBb function as it acts as a pattern recognition molecule for binding of the convertase complex to microbial surfaces and stabilizes covalently bound C3bBb, greatly extending its active lifetime (18, 19). Salivary inhibitors from ixodid ticks are effective complement inhibitors that function by scavenging properdin, thereby preventing its binding with convertase complexes (20, 21).

Natural inhibitors of the complement pathway and their mechanisms of inhibition are of continuing interest. Several diseases, including age-related macular degeneration (AMD), atypical hemolytic uremic syndrome (aHUS), and paroxysmal nocturnal hemoglobinuria (PNH) occur as a result of unregulated complement activation, and drugs targeting the complement pathway are currently being studied or are in use for their treatment (22, 23). Previously, we isolated albicin, a member of the salivary SG7 protein family from females of the malaria mosquito *An. albimanus*, which inhibits activation of the AP in human serum as measured by lysis of rabbit erythrocytes, blocks the cleavage of C3 and factor B in serum, and binds specifically to the C3bBb complex (14). On surface plasmon resonance (SPR) surfaces of properdin, albicin stabilizes binding of the complex but also prevents C3 cleavage by reconstituted solution phase C3bBb in the absence of properdin (14). It does not directly inhibit the enzymatic activity of factor D, nor does it block the cleavage of factor B. Salivary gland extracts of a second *Anopheles* species, *An. freeborni*, also inhibit the activation of complement in serum (14). In this study, we describe the structures of albicin and its orthologs SG7.AF (from *An. freeborni*) and anophensin (from An. stephensi), characterize the binding and mechanism of AP inhibition by these inhibitors, and determine a role for properdin in modulating the inhibitory activity of SG7.AF. We also demonstrate that while the SG7 protein family is distributed throughout the species of *Anopheles*, not all variants possess strong anti-AP activity.

## Results

### SG7.AF blocks activation of the AP

**Figure 1:**
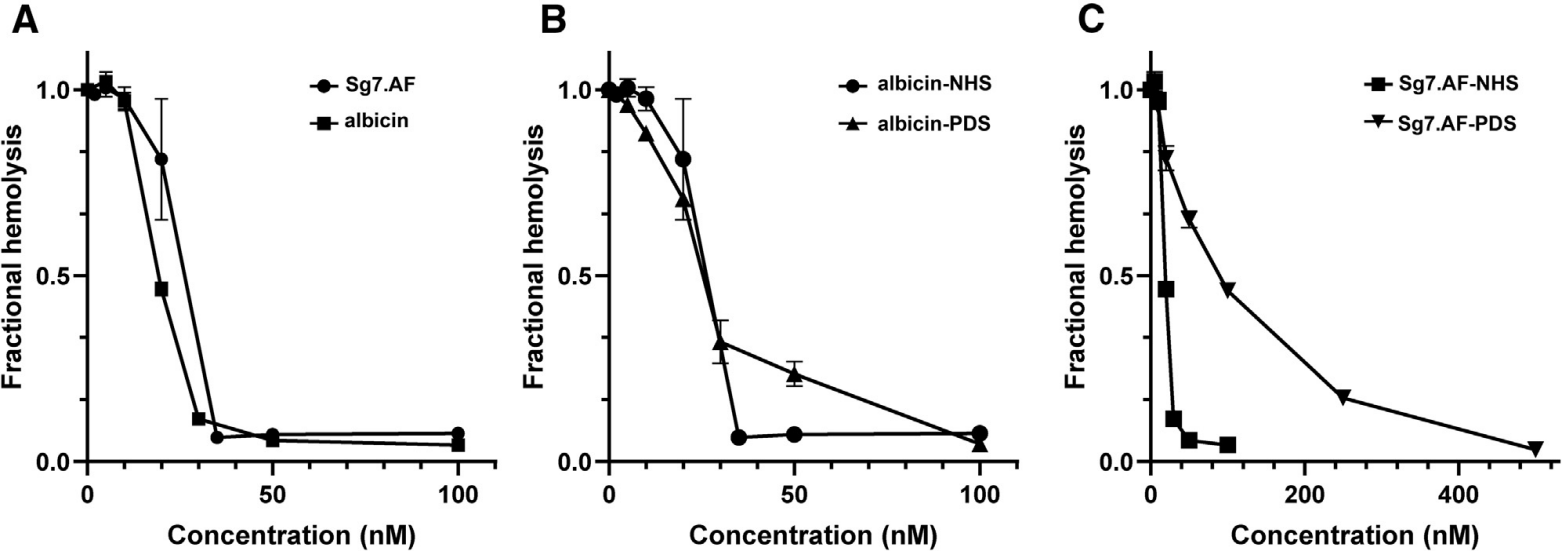
**Inhibition of AP activation by mosquito-derived inhibitors as measured by erythrocyte lysis:***A*, inhibition of AP-mediated hemolysis of rabbit erythrocytes by SG7.AF (*circles*) and albicin (*squares*) in NHS (diluted 1:20). *B*, inhibition of AP-mediated hemolysis of rabbit erythrocytes by albicin in NHS (diluted 1:20, *circles*) and PDS (diluted 1:2, *triangles*). *C*, inhibition of AP-mediated hemolysis of rabbit erythrocytes by SG7.AF in NHS (*squares*) and PDS (*triangles*).

Complement activation can be quantified by observing the lysis of rabbit erythrocytes when incubated with human serum. These cells consistently activate the AP in human serum and are considered as surrogates for foreign cells encountered during infection *in vivo*. Albicin was previously shown to prevent activation of the AP in the lysis assay using human serum. The protein was equally effective in normal human (NHS) and properdin-depleted (PDS) serum indicating that properdin is not involved in its inhibitory mechanism (Fig. 1, *A*–*B*). We performed the same assays with recombinant SG7.AF from *An. freeborni* and found it to block erythrocyte lysis effectively in NHS but less potently in PDS (Fig. 1, *A* and *C*). The IC_50_ value for SG7.AF was similar to that of albicin in NHS but increased by sixfold in PDS suggesting a role for properdin in its mechanism of action (Fig. 1, *B*–*C*). Identically to albicin, SG7.AF showed no ability to inhibit the CP (Fig. S1*A*). The fact that SG7.AF remained inhibitory, albeit less so, in the absence of properdin indicates that it does not function simply as a scavenger of properdin, but rather is made more potent in its anti-C3bBb activity by properdin.

**Figure 2:**
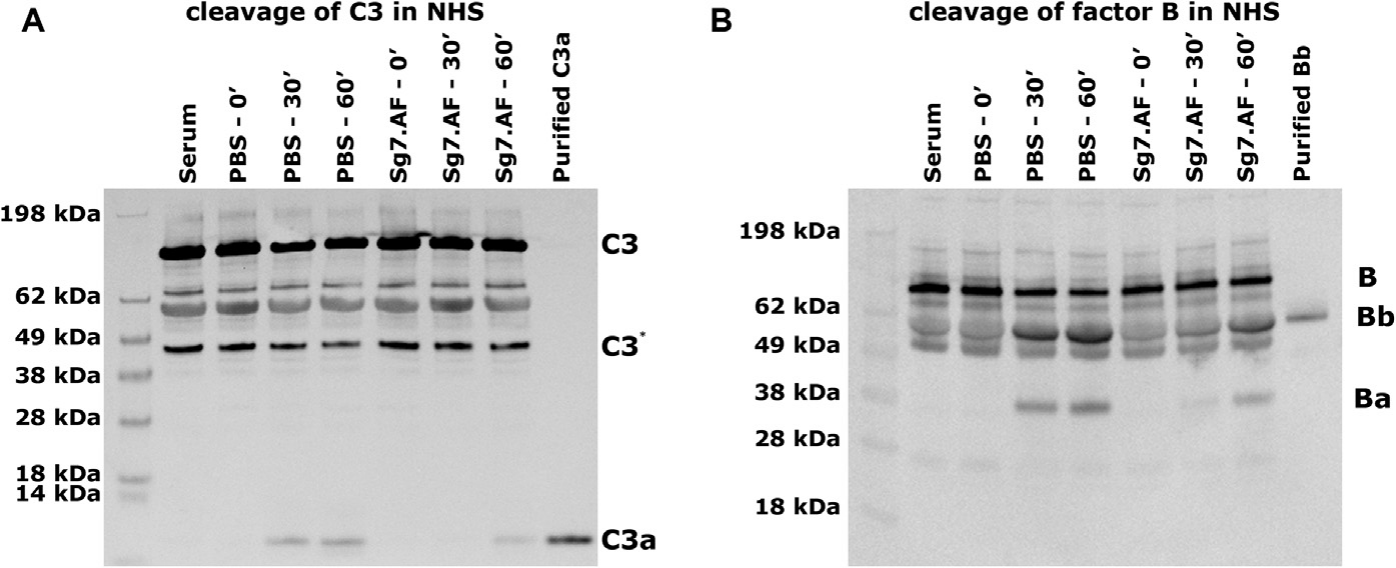
**Evaluation of factor B activation and C3bBb activity in NHS in the presence of SG7.AF:** 5 μl of the supernatant from the AP-mediated hemolysis assay in the presence of inhibitors was collected at 0, 30, and 60 min. Proteins were separated on a 10% NuPage gel and transferred to nitrocellulose membranes. *A*, NHS C3bBb activity was evaluated for C3a release using anti-C3a (1:10,000). A C3 degradation product that appears after heating SDS-PAGE samples is labeled C3∗. *B*, activation of factor B (B) was evaluated by the formation of factor Ba (Ba) and factor Bb (Bb) using anti-factor B (1:10,000).

**Figure 3:**
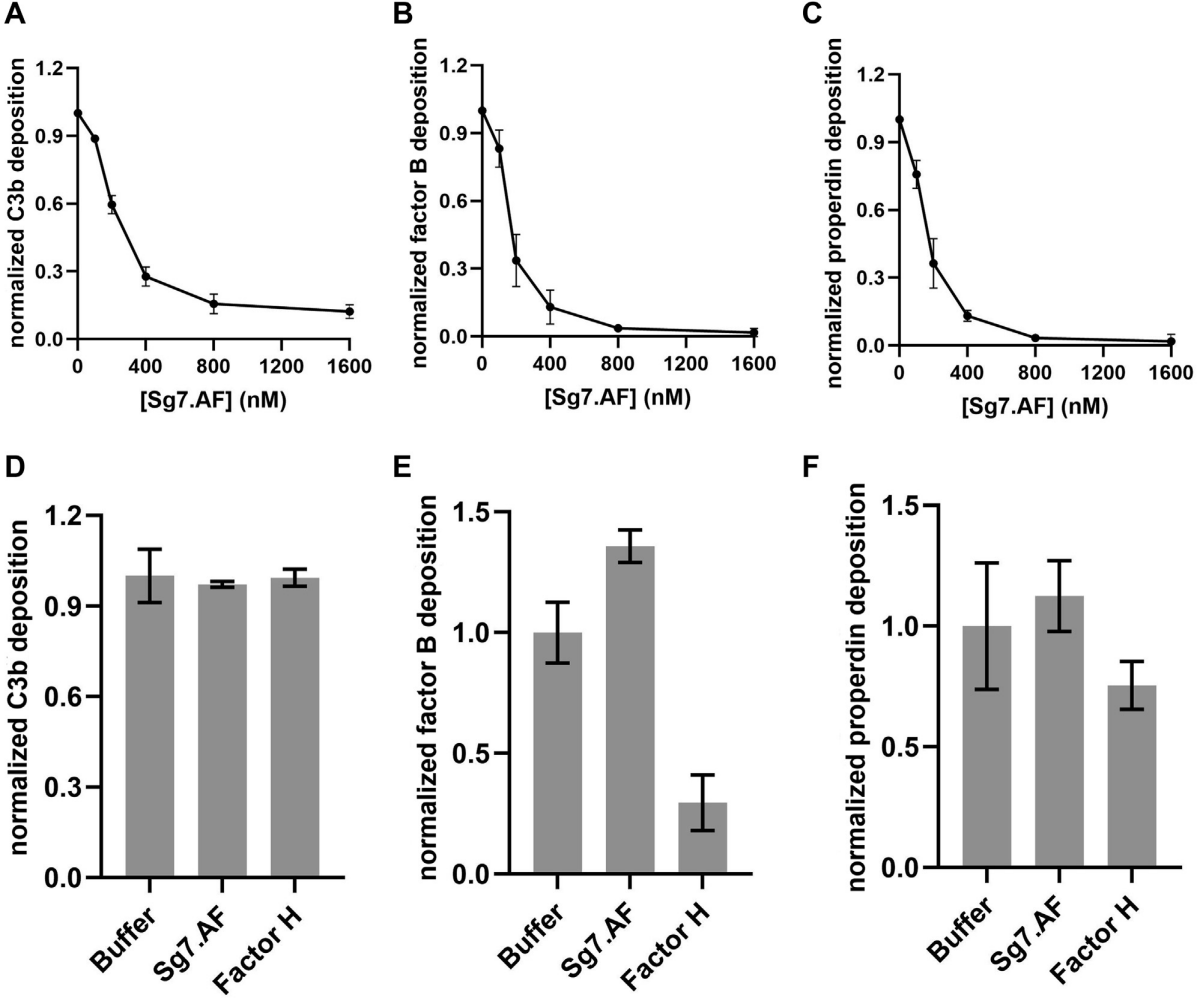
**Deposition and displacement of C3b, factor Bb, and properdin in the presence of SG7.AF:** Agarose-coated plates were incubated with NHS (20%) and different concentrations of SG7.AF at 37 °C for 30 min and probed with (*A*) anti-C3 (1:5000), (*B*) anti-factor B (1:200), or (*C*) anti-properdin (1:200). Each experiment was run twice and replicated three times in each run. The data were normalized to the zero concentration value and the points represent means ± standard deviation. For displacement assays, plates were incubated with NHS (20%) for 30 min at 37 °C followed by incubation with SG7.AF (800 nM) or factor H (10 μg) for 30 min at 37 °C and probed with (*D*) anti-C3 (1:5000), (*E*) anti-factor B (1:200), or (*F*) anti-properdin (1:200). Wells treated with serum not containing SG7.AF were used as positive control, and wells probed in the absence of both serum and SG7.AF were used as negative control. Each experiment was run twice and replicated three times in each run. The data were normalized to the buffer value, and the bars represent the mean ± standard deviation. The lack of effect of factor H on C3b dissociation is consistent with covalent attachment to the agarose surface.

**Figure 4:**
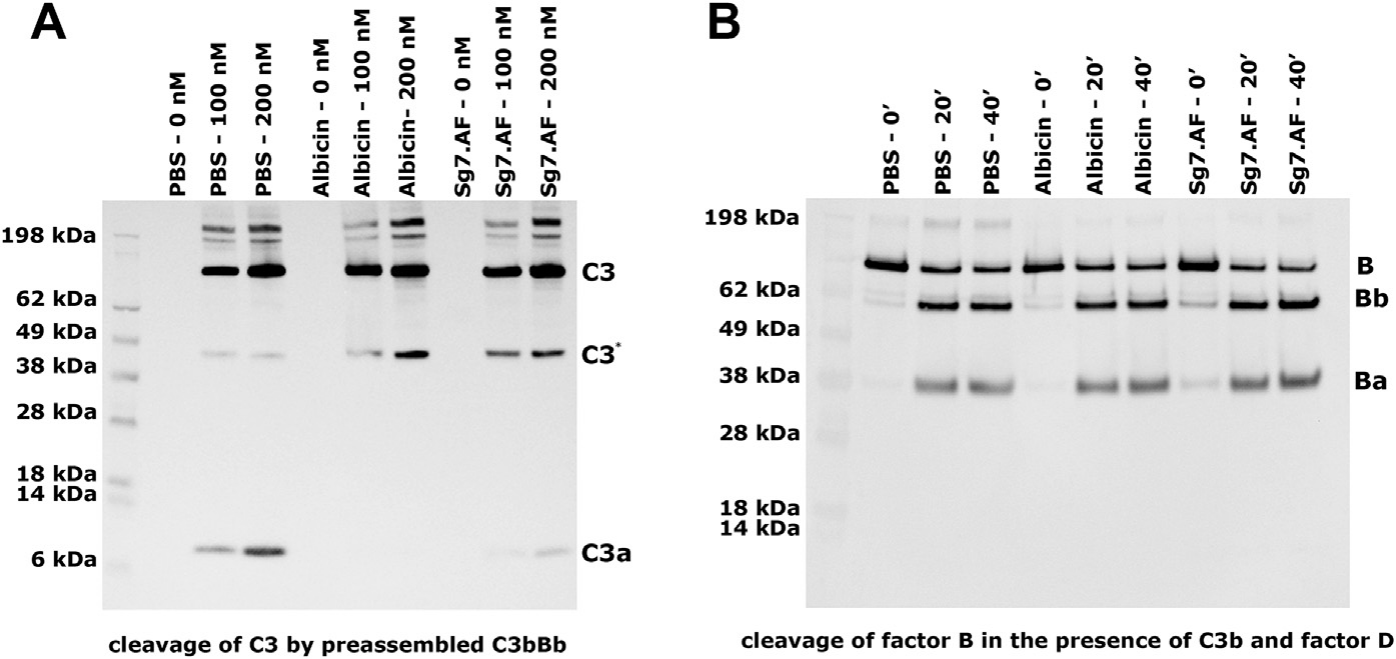
**Effect of SG7.AF and albicin on the activity of reconstituted C3bBb and the activation of C3bB by factor D.***A*, C3bBb was formed *in vitro* by incubation of C3b (200 nM), factor B (100 nM), and factor D (50 nM) at room temperature for 2 min followed by addition of EDTA (5 mM). The resulting C3bBb complex was incubated with C3 (0, 100, or 200 nM) in the presence of SG7.AF or albicin (2 μM) for 20 min at room temperature, separated on a 10% NuPAGE gel, and transferred to a nitrocellulose membrane. C3bBb activity was evaluated by the formation of C3a using anti-C3a (1:10,000). A C3 degradation product that appears after heating SDS-PAGE samples is labeled C3∗. *B*, C3b (200 nM), factor B (100 nM), and factor D (50 nM) were incubated in the presence or absence of SG7.AF or albicin (2 μM) for 0, 20, and 40 min at 37 °C. Proteins were separated on a 10% NuPAGE gel and transferred to a nitrocellulose membrane. Cleavage of factor B (B) into factor Ba (Ba) and Bb was evaluated using anti-factor B (1:10,000).

Mechanistic differences between properdin-independent and -dependent SG7 inhibitors were probed by comparison of SG7.AF with albicin in a variety of additional assays. In supernatants of erythrocyte lysis assay preparations, the cleavage of C3, as measured by the appearance of its cleavage product C3a on western blots, was inhibited in the presence of SG7.AF as was the cleavage of factor B, indicating that SG7.AF interferes with the production of the alternative C3 convertase and prevents amplification of complement activation in a manner similar to albicin (Fig. 2). Like albicin, SG7.AF prevents C3b, factor B, and properdin deposition from serum onto agarose-coated plates, which are considered to mimic the foreign surfaces activating complement *in vivo* by supporting covalent linkage of C3b through hydroxyl groups on agarose (16). This verifies that SG7.AF blocks AP activation at the point of the C3bBb complex or before (Fig. 3, *A*–*C*). No significant binding of individual protein components of the AP to plate bound SG7.AF was observed when measured by ELISA, suggesting that the inhibitor binds to the assembled complex rather than a single protein (Fig. S1*B*). Additionally, SG7.AF did not induce dissociation of immobilized C3bBb complexes preassembled on agarose plates, while addition of factor H causes release of factor B and properdin (Fig. 3, *D*–*F*). This demonstrates that, like albicin, SG7.AF does not disrupt the integrity of the complex in the manner of factor H/factor I or other endogenous complement regulators. SG7.AF did inhibit the enzymatic activity of reconstituted C3bBb complexes in solution as measured by the appearance of C3a after incubation of C3 with preassembled C3bBb, but was less potent than albicin, which completely blocks the appearance of C3a on western blots (Fig. 4*A*). Like albicin, SG7.AF did not block the activation of factor B by factor D (conversion of C3bB to C3bBb) demonstrating that it targets the C3bBb complex directly, rather than inhibiting the serine protease responsible for its activation (Fig. 4*B*). Together, these data suggest that SG7.AF acts similarly to albicin in that it inhibits the catalytic activity of the C3bBb complex by directly interacting with it. However, it is more potent in the presence of properdin than in its absence.

### Albicin and SG7.AF enhance accumulation of C3bBb on properdin SPR surfaces

**Figure 5:**
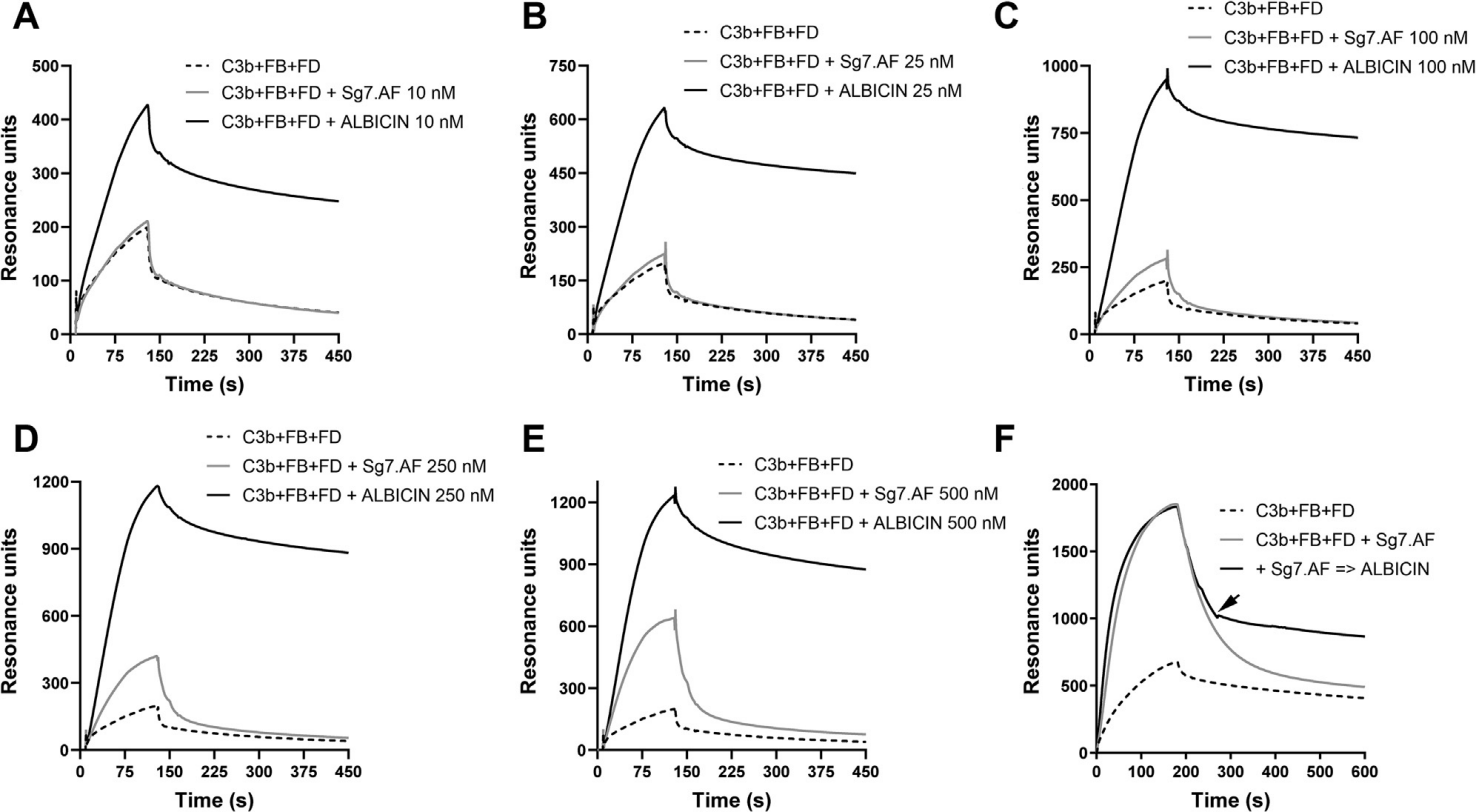
**Effect of SG7.AF and albicin on C3bBb assembly on a SPR surface of immobilized properdin (5000 RU).***A–E*, C3b (20 μg/ml), factor B (FB, 20 μg/ml), and factor D (FD, 2 μg/ml) were injected in the absence or presence of different concentrations of SG7.AF or albicin at a buffer flow rate of 30 μl/min for 2 min. *F*, exchange of albicin and SG7.AF in C3bBb bound on a properdin surface. Albicin was injected for 60 s (beginning at arrow) during the dissociation phase following assembly of the properdin-bound SG7.AF-C3bBb complex. Concentrations of SG7.AF and albicin were 1 μM. The plots are representative of results from two properdin surfaces.

In addition to its role in stabilizing the binding of C3b with factor B and Bb, properdin binds to cell surfaces and acts as a matrix for assembly of the C3bBb complex (19). Properdin-coated SPR surfaces mimic this condition and support the assembly of C3bB and C3bBb in a similar manner (24). We have shown previously that albicin enhances rather than blocks the accumulation of C3bBb on immobilized properdin suggesting that the inhibitory mechanism involves a strengthening of the interaction of the complex with the surface. When injected along with C3b, factor B, and factor D, SG7.AF and albicin both cause an enhanced accumulation of C3bBb on the surface, but SG7.AF was required at higher concentrations, indicating a lower-affinity binding interaction for this inhibitor (Fig. 5*A*). SG7.AF alone, C3bB, C3bB-SG7.AF, and C3b-SG7.AF showed little or no interaction with the surface (Fig. S2). The large increase in accumulation of C3bBb on the surface observed in the presence of inhibitors suggests a substantial increase in the overall affinity of the inhibited C3bBb complex for the properdin surface relative to C3bBb alone.

Dissociation of the complex from the surface exhibited biphasic kinetics with the overall dissociation of the SG7.AF-bound complex being substantially more rapid than the albicin-bound complex (Fig. 5*E–F*). The dissociation data at inhibitor concentrations of 1 μM were fit to a double exponential decay function. The fast phase for release of both the SG7.AF- and albicin-bound complexes exhibited a rate constant of 0.02 s^−1^, but with proportional amplitudes of 0.8 for SG7.AF-bound and 0.2 for albicin-bound complexes indicating that the albicin-bound complex was present mainly as a stable, slow-dissociating form while the SG7.AF complex was present mainly as a rapidly dissociating form. The slow phase rate constant for both SG7.AF and albicin complexes was 0.003 s^−1^. When albicin was injected after deposition of C3bBb-SG7.AF on the chip surface, the dissociation rate for the complex was reduced to a value similar to that of the bound C3bBb-albicin complex indicating that SG7.AF and albicin are rapidly exchanged in properdin-bound C3bBb and that inhibitor binding regulates the decay rate (Fig. 5*F*). If 1:1:1 stoichiometry is assumed for C3b, factor Bb, and SG7.AF in the bound complex, C3b would account for 70% of the complex mass. The species dissociating in the fast phase (80% of the complex mass) must therefore contain C3b and is most likely the entire monomeric C3bBb complex being released from the properdin surface after dissociation of the inhibitor.

### Chromatographic analysis of the albicin-bound C3bBb complex.

**Figure 6:**
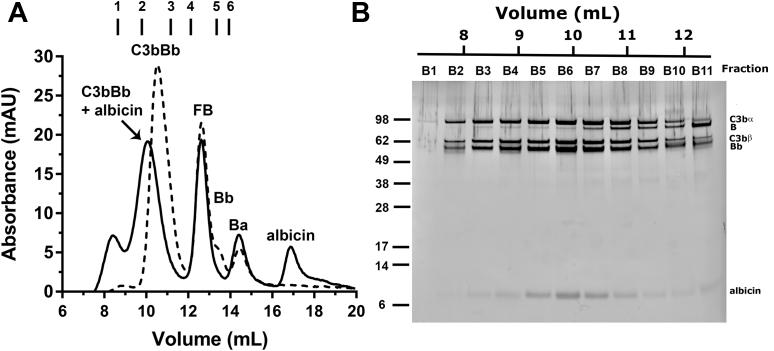
**Gel filtration chromatography of the albicin C3bBb complex.***A*, C3b, factor B, and factor D were incubated in the presence (solid line) or absence (dotted line) of albicin for 15 min at room temperature in HBS containing 5 mM NiCl_2_. The component molar ratio was 1:2:1:5 C3b: factor B: factor D: albicin. The mixture was passed through a Superdex 200 10/300 column, and the absorbance was measured at 280 nm. Marks above the chromatogram are the positions of molecular weight standards: 1 = thyroglobulin (669 kDa), 2 = ferritin (440 kDa), 3 = C3b (176 kDa), 4 = aldolase (158 kDa), 5 = conalbumin (75 kDa), 6 = ovalbumin (44 kDa). *B*, the fractions were analyzed by SDS-PAGE and visualized by silver staining. Bar above the gel represents the retention volume of the fractions in the chromatogram of panel *A*.

The increased accumulation of the albicin–C3bBb complex on properdin SPR surfaces suggested that the inhibitor may induce oligomerization in the manner of the staphylococcal complement inhibitor SCIN, whose binding results in dimerization of C3bBb (25). We assessed the oligomeric state of the albicin-inhibited complex observed in SPR experiments using gel filtration chromatography after coincubation of C3b, factor B, and factor D in the presence and absence of albicin and nickel ion, which is known to enhance the binding of C3b with factor B (25). Albicin markedly reduced the retention volume for complex elution, indicating an increased molecular mass for the inhibited complex beyond that attributable to addition of the inhibitor alone (Fig. 6*A*). Based on chromatographic data from a series of standards, the complex has a molecular weight 360 kDa, while the calculated mass of the dimeric complex is 499 kDa, suggesting a C3bBb–albicin dimer is present that partially dissociates during chromatography (Fig. 6*A*, Fig. S3). Nevertheless, the monomeric C3bBb complex (250 kDa) formed in the absence of albicin appeared near its predicted elution volume and showed distinct separation from the inhibited complex (Fig. 6*A*). Examination of chromatographic fractions by sodium dodecyl sulfate-polyacrylamide gel electrophoresis (SDS-PAGE) also showed albicin to be present along with C3b and factor Bb in the fractions corresponding to the UV absorbance peak of the inhibited complex (Fig. 6*B*, Fig. S3).

### The crystal structures of albicin and SG7.AF

**Figure 7:**
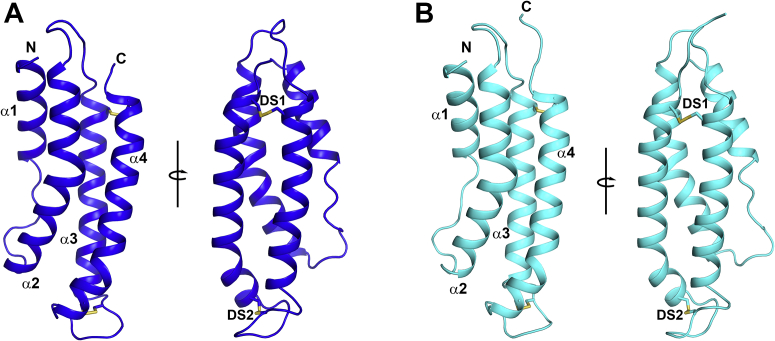
**Crystal structures of albicin and SG7.AF.***A*, Ribbon diagram of albicin with the left and right panels being related by rotation of approximately 90° around the axis shown. Alpha helical elements are labeled α1-α4 and the N- and C-termini are indicated. Cysteine residues are shown in stick representation with sulfur colored in yellow. The two disulfide bonds are labeled DS1 and DS2. *B*, structure of SG7.AF with the two panels showing views comparable to those in panel *A*.

**Figure 8:**
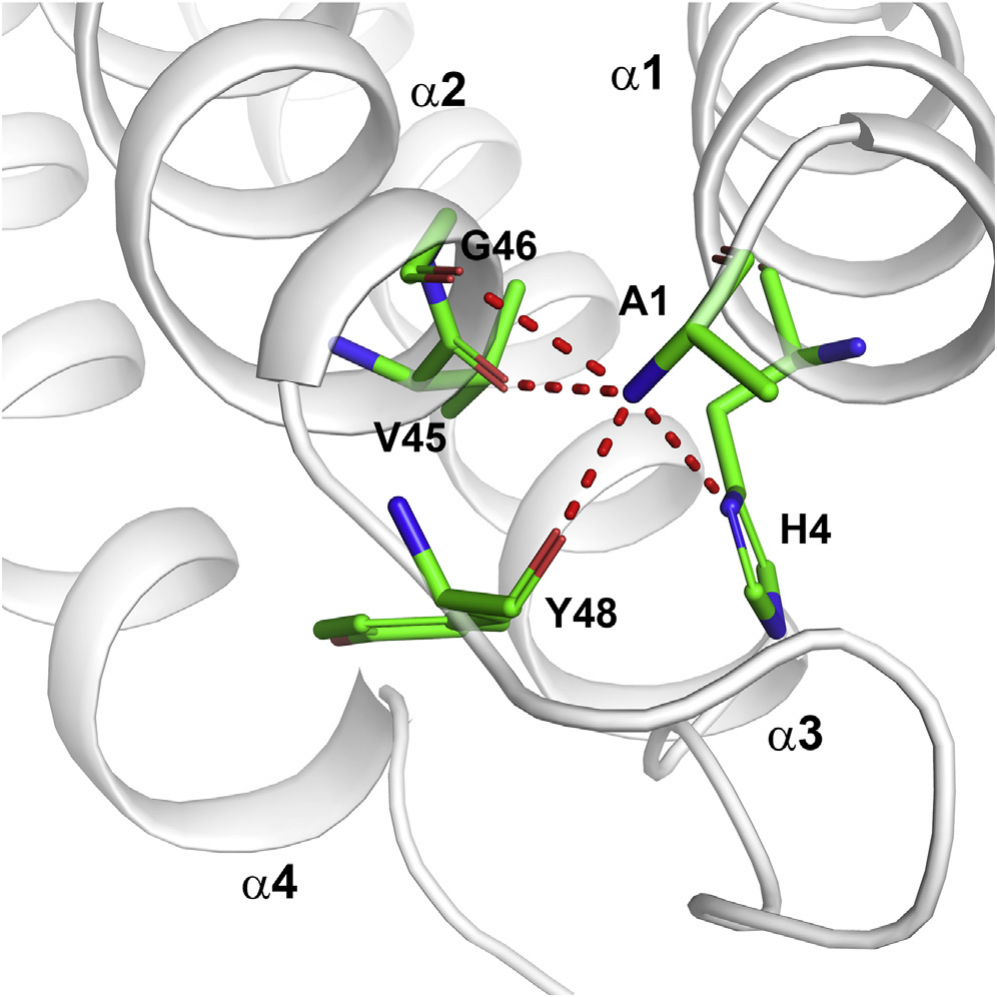
**N-terminal hydrogen bonding network of albicin and SG7.AF.** Ribbon diagram of albicin with N-terminal hydrogen bonding network shown in stick representation. Side chains are shown in *green* with nitrogen shown in *blue* and oxygen in *red*. Hydrogen bonds are shown as *red dashed* lines. Helical elements and residues participating in hydrogen bonds are labeled.

The structure of albicin consists of a bundle of four α helices stabilized by two disulfide bonds (Fig. 7*A*, Table 1). Helix α1 extends from the N-terminus to Thr 14 and is followed by a section of random coil that extends to Lys 22. Helix α2 extends from Ser 23 to Gly 47, and the loop linking α2 and α3 extends from Tyr 48 to Ser 55 followed by α3 extending from Ser 23 to Gly 47 (Fig. 7*A*). The loop connecting helices α3 and α4 extends from Ser 78 to Ser 88, with α4 extending from Val 89 to the C-terminus. The two disulfide bonds link α3 and α4 with Cys 58 forming a disulfide with Cys 113 and Cys 81 linking with Cys 91. The N-terminal amino group of albicin participates in numerous intramolecular electrostatic interactions that appear to be important in stabilizing the overall structure, including three hydrogen bonds with residues forming the turn linking α2 and α3 (Fig. 8). The carbonyl groups of Val 45, Gly 46, and Tyr 48 form hydrogen bonds with the N-terminal amino group as does ND1 of the imidazole group of His 4 in α1. These interactions may be important for the positioning of the helical elements and stabilization of the helical bundle. Structural searches using DALI (26) reveal similar structural arrangements in other proteins, especially as portions of larger molecules. The level of amino acid identity in these structures is very low (≤10 % amino acid identity), suggesting that the simple antiparallel four-helix bundle may have evolved independently in the SG7 group.

**Table 1:** Data collection, phasing and refinement statistics for albicin, SG7.AF, and anophensin (Anoph)

Crystal	Albicin	SG7.AF	Anoph-Se	Anoph
Resolution (Å)	52–1.55	41–1.4	50–2.7	82–2.31
Beamline	22-ID	22-ID	22-ID	22-ID
Wavelength (Å)	0.9184	1.0000	0.9791	1.0000
Completeness (total/high-resolution shell)	96.3/53.0	99.2/88.6	99.8/99.0	100/100
Average redundancy (total/high-resolution shell)	11.1/3.6	12.9/4.5	3.5/3.7	7.9/6.9
R_merge_ (total/high-resolution shell, %)	7.8/44.9	5.1/22.2	6.9/22.7	5.9/57.7
CC_1/2_ (total/high-resolution shell)	99.8/84.2	99.9/95.2	99.9/99.0	99.7/95.1
I/sigI (total/high-resolution shell)	19.1/2.8	31.4/5.3	14.0/9.8	19.4/4.1
Observed reflections	757,079	425,154	94,980	142,373
Unique reflections	67,109	32,895	11,471	18,055
Space group	P2_1_2_1_2	P4_1_2_1_2	P2_1_2_1_2	P2_1_2_1_2
Unit cell dimensions (Å)				
A	56.68	85.98	67.64	67.28
B	137.68	85.98	82.14	82.36
C	61.14	46.08	71.35	71.73
α, β, γ (°)	90	90	90	90
No. of Se/Br sites	8		16	
FOM (Phenix autosol)			0.39	
Contrast (ShelxE)	0.44			
Refinement				
Total non-H protein atoms	2832	1013		2824
Total non-H solvent atoms	429	212		
RMS deviations				
Bond lengths (Å)	0.006	0.005		0.007
Bond angles (°)	0.806	0.838		0.86
Mean B factors (Å^2^)				
Protein	19.0	17.4		61.8
Solvent	26.5	31.9		
Bromide	34.4			
MolProbity analysis				
Ramachandran plot (favored/allowed, %)	97.7/99.4	98.3/100		95.0/100
Clashscore	0.88	4.3		4.1
Rotamer outliers (%)	0.0	0.87		1.4
Coordinate error ML (Å, Phenix)	0.16	0.14		0.34
R_cryst/_R_free_	0.18/0.20	0.18/0.19		0.23/0.26

The backbone structure of SG7.AF is very similar to that of albicin (RMSD = 0.51 Å), with the lengths of the helices and positions of the cysteine residues being essentially the same (Fig. 7*B*, Table 1). SG7.AF also contains an N-terminal structure identical to that of albicin where the amino group is stabilized by means of the same hydrogen bonding network. At the amino acid level, all are approximately 50% identical, and the identical residues are distributed throughout the four helical elements (Fig. S4). Interestingly, the structure shows similarities in its size and dimensions with SCIN, the staphylococcal inhibitor that also causes dimerization of the C3bBb complex (25, 27). The SCIN family structure consists of a bundle of three antiparallel α helices with a long dimension of ∼41 Å and an orthogonal short dimension of ∼14 Å, while the four-helix bundle of albicin has dimensions of 50 and 21 Å. SCIN recognizes two binding sites on C3b that are used to link monomers into a symmetrical dimeric structure. Each SCIN binds one site on each monomer simultaneously, and two SCIN molecules act to form the dimeric structure. SPR and chromatographic evidence suggest that a similar mechanism of action for albicin is possible, but details of these interactions remain to be shown.

### Anophensin, an SG7 ortholog from An. stephensi

**Figure 9:**
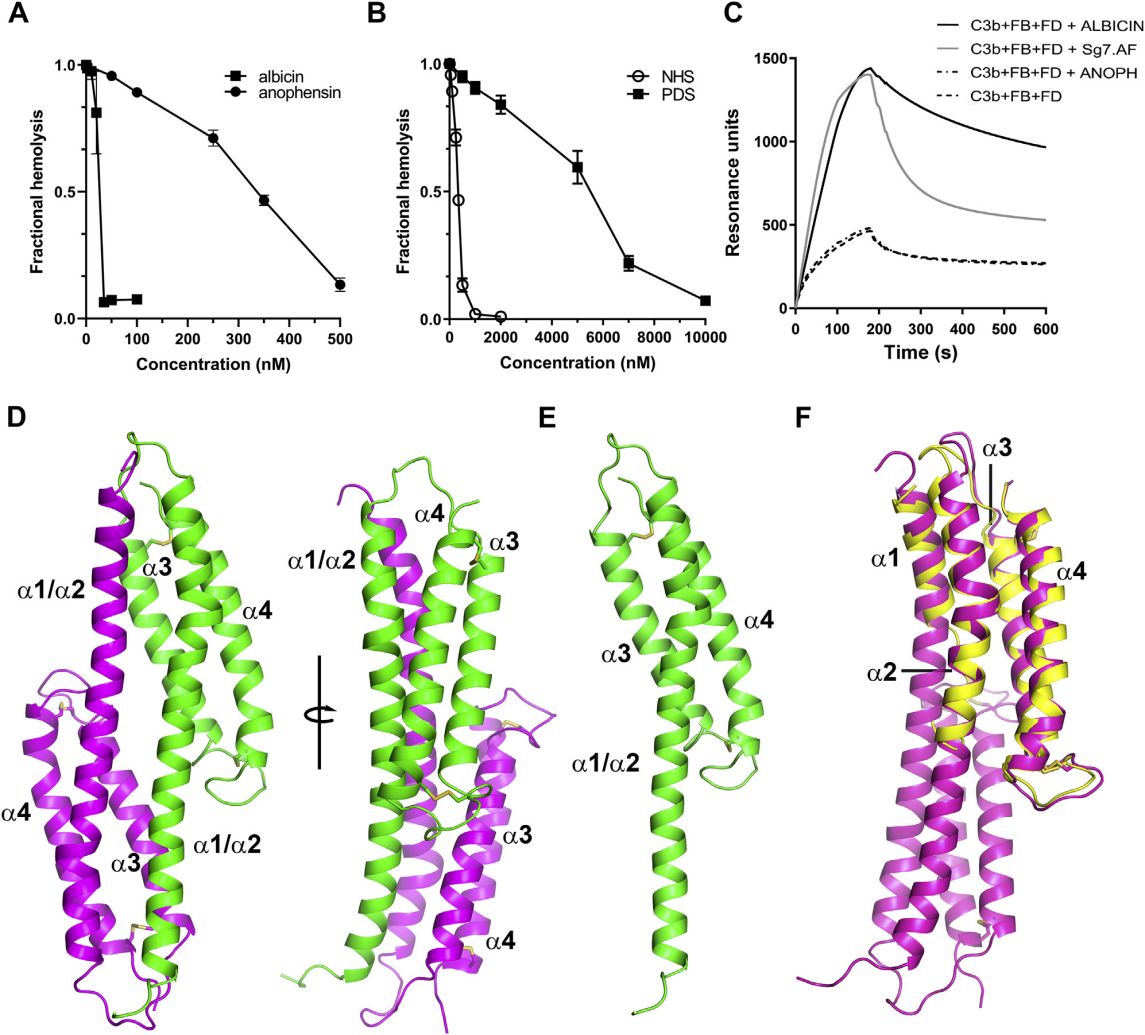
**Crystal structure and anti-AP activity of anophensin.***A*, inhibition of AP activation in NHS by albicin (*squares*) and anophensin (*circles*) as measured by hemolysis of rabbit erythrocytes. *B*, inhibition of AP activation in NHS (*open circles*) or PDS (*squares*) by anophensin. *C*, comparison of accumulation of C3bBb on properdin surfaces in the presence of SG7 as measured by SPR. The concentration of the convertase components was 56.8 nM C3b, 107.5 nM factor B (FB), 41.7 nM factor D (FD), and the inhibitor concentrations were 1 μM (ANOPH = anophensin). *D*, structure of the domain-swapped dimer form of anophensin extended in length by three amino acids. The right and left models are related by rotation of approximately 90° around the axis shown. The two chains are colored *magenta* and *green*. The helical elements are labeled, and cysteine residues are shown in stick representation with sulfur shown in *yellow*. *E*, monomer of anophensin extended by three amino acids. *F*, Dimer of anophensin (*magenta*) superimposed with the monomeric albicin model (*yellow*) to highlight domain swapping.

Salivary gland extracts from species of the *Anopheles* subgenus *Cellia* are known to possess considerably less AP inhibitory activity than *An. albimanus* (subgenus *Nyssorynchus*) or *An. freeborni* (subgenus *Anopheles*) extracts despite the fact that all investigated species contain SG7 orthologs (14, 28). Anophensin, the albicin ortholog from An. stephensi, has been shown to inhibit the contact pathway of coagulation through binding with factor XIIa and high-molecular-weight kininogen (29). We evaluated the AP inhibitory activity of recombinant anophensin in the erythrocyte lysis assay and found it to be much less potent than albicin or SG7.AF with an IC_50_ value of approximately 350 nM in NHS and 6 μM in PDS (Fig. 9, *A*–*B*). At a concentration of 1 μM, the protein also did not detectably stabilize assembly of C3bBb on a properdin SPR surface as do albicin and SG7.AF (Fig. 9*C*). Nevertheless, the high sequence identity and nearly identical chromatographic characteristics of the recombinant protein suggested a close structural similarity to albicin and SG7 (Fig. S4).

We obtained crystals of a variant of anophensin containing three additional amino acids at its N-terminus. This variant exhibits a reduced retention volume in gel filtration chromatography suggesting oligomerization (Fig. S5). Molecular replacement, using albicin as a search model, did not produce an obvious solution suggesting that the structures of the two proteins differed significantly. The structure was then determined experimentally using a selenomethionine derivative and found to be significantly rearranged in that helix α1 is moved away from the rest of the helical bundle and forms part of a single extended alpha helical structure running from the N-terminus to the C-terminal end of α2 (Fig. 9*D*, Table 1). This presumably occurs due to loss of the stabilizing effect of the N-terminal hydrogen bonding network described above as a result of lengthening of the peptide and changing the position of the free N-terminal amino group. In the crystal, anophensin occurs as a symmetrical dimer in a domain-swapped arrangement where a number of the amino acid interactions in albicin between α1 and the rest of the bundle are retained but are now intermolecular. The extended coil region linking α1 and α2 also becomes helical, creating several new intermolecular interactions that may stabilize a dimeric form of the protein in solution (Fig. 9*F*). Dimerization in solution may account for the decreased gel filtration retention volume and reduced activity of this variant (Fig. S5).

The structural changes observed in this lengthened anophensin could also be relevant with regard to the SG7-like mosquito salivary protein group known as the 30 kDa antigens represented by the anopheline antiplatelet protein (AAPP) from *An. stephensi* and aegyptin from *Ae. aegypti* (30, 31). These contain a C-terminal region homologous to the SG7 proteins and a long, low-complexity N-terminal sequence of at least 104 residues that is predicted to have an intrinsically disordered structure. They prevent platelet activation by binding to exposed collagen at the feeding site, thereby blocking interaction with platelet receptors. The active portion of AAPP is contained in the C-terminal, SG7-like region, with the N-terminal low-complexity sequence apparently being dispensable for interaction with collagen (32). A published crystal structure of the C-terminal part of active region of AAPP corresponding to α3 and α4 of SG7 proteins shows a similar hairpin arrangement and the same disulfide bonding pattern as albicin. The structure does not contain the region homologous to α1 and α2 that is essential for activity. Nevertheless, the anophensin structure with its extended N-terminus suggests that the α1-α2 region of the 30 kDa family might be unable to form the N-terminal hydrogen bonding network seen in albicin and SG7.AF and possibly assume a partially unfolded structure that could be relevant for the interaction with collagen.

## Discussion

Various lines of evidence show that complement activation is deleterious to feeding and the digestion of blood by mosquitoes. Complement is activated within minutes in the midgut, but damage to tissue is mitigated by the presence of anticomplement factors. *An. gambiae* binds host factor H through specific receptors on the gut surface, leading to inactivation of complement in the blood meal and protection of digestive tissues (33). Salivary inhibitors may also assist in this process since a significant proportion of the secreted saliva is ingested along with blood. However, the presence of inhibitors in saliva makes it more likely that they act primarily to facilitate the ingestion of blood. At the host feeding site, complement products modulate wound-induced inflammatory responses in the skin that hinder blood intake and elicit host defensive behaviors. The anaphylatoxins C3a and C5a produced by cleavage of C3 and C5 are potent proinflammatory molecules that induce immediate responses relevant to mosquito feeding (34, 35, 36). Injection of C3a and C5a into the skin produces wheal and flare reactions that appear quickly enough to affect feeding (36). Plasma extravasation, swelling, itching, and pain associated with this reaction may interfere with the physical process of taking blood and cause the host to react to the presence of the insect. Much of this effect is mediated through activation and degranulation of mast cells. C5a is more potent than C3a as a skin mast cell agonist, but both are quite active (34), and blockade of complement activation at the alternative C3 convertase would eliminate production of both. Local trauma caused by piercing of the skin with the mosquito mouthparts may be sufficient to stimulate complement activation since apoptotic cells and the contents of damaged cells are known to be activators of the AP. To combat these defenses, *Anopheles* mosquitoes produce inhibitors belonging to the SG7 protein family that block complement at the alternative C3 convertase, C3bBb. Early recognition of this central complex apparently allows blockade of the pathway with the small amount of protein present in saliva that is diluted into a much larger volume of blood.

**Figure 10:**
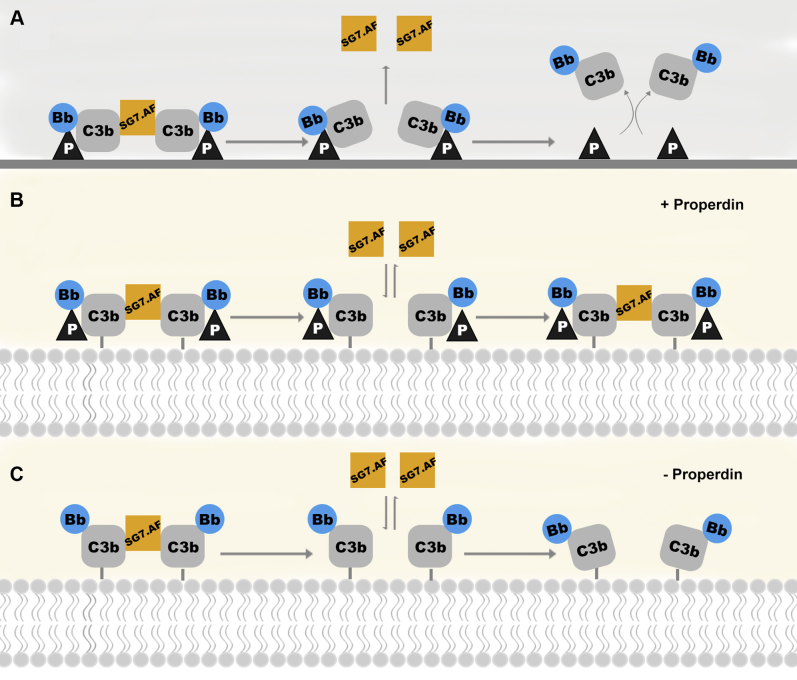
**Mechanistic models for inhibition of the C3bBb complex.***A*, SG7.AF binding to C3bBb assembled on an immobilized properdin SPR surface. Two C3bBb complexes (left) bound to immobilized properdin assemble as a dimer linked by SG7.AF (two molecules are assumed, but only one is visible). The dimeric complex is bound to two properdin molecules. After dissociation of SG7.AF (center), monomeric C3bBb is bound to properdin at only one site and dissociates from the surface (right). *B*, inhibited C3bBb dimers covalently linked to the erythrocyte membrane are stabilized by properdin (left). Bound properdin maintains the dimeric structure (center) allowing rebinding of inhibitor. *C*, in the absence of properdin, the dimeric structure forms (left) are less stable and more mobile (center) reducing the affinity for the inhibitor and allowing activation. C3bBb becomes catalytically active after dissociation of the inhibitor.

Here we examined the functions of SG7.AF and albicin, two inhibitors of the alternative C3 convertase found in the saliva of female *Anopheles* mosquitoes. The two proteins are very similar in structure and mechanism of action, but SG7.AF binds C3bBb with lower affinity than albicin and is more active in the presence of properdin than in its absence. Properdin acts as a pattern recognition molecule serving as a substrate for complex assembly as well as a stabilizing factor for covalently attached C3bB and C3bBb (Fig. 10). It binds to the outer surfaces of cells and promotes noncovalent binding of C3b and binding of additional C3b molecules through polyvalent interactions (19) (Fig. 10). Covalently bound C3bB and C3bBb complexes are also stabilized by properdin, which increases the apparent affinity of factor B (and Bb) interaction and lengthens the active lifetime of C3bBb by up to a factor of 10 (18) (Fig. 10). C3bB and C3bBb also bind properdin in solution where its stabilizing effects are similar to those seen with surface-bound complexes (37). It has been suggested that binding of C3bBb to properdin-coated SPR surfaces is analogous to the noncovalent association of C3bBb to properdin bound at the surfaces of cells (24).

We found that individual C3bBb components, including properdin, do not bind SG7.AF immobilized on ELISA plates, but the inhibitor promotes a synergistic accumulation of C3bBb assembled on immobilized properdin SPR surfaces, reflecting an increase in the affinity of the inhibited complex for the surface. Here and previously, we have also shown that albicin produces a similar effect (14). Furthermore, gel filtration analysis shows that albicin induces formation of an oligomeric C3bBb complex containing bound inhibitor. Oligomerization could explain the increased affinity for properdin surfaces as simply being due to an increase in the number of properdin interaction sites (Fig. 10). The crystal structure of C3bBb in complex with SCIN, a staphylococcal AP inhibitor, contains a dimer having twofold symmetry held together by two SCIN molecules, which make bridging contacts between the C3bBb monomers (25). An analogous structure for C3bBb inhibited by albicin and SG7.AF would have two times the number of contacts with the properdin surface as the monomer, explaining the enhanced interaction with the properdin surface.

Recruitment of solution-phase C3b by properdin bound to covalently linked C3b on microbial surfaces may help to orient C3bBb complexes in inhibited oligomers, potentially explaining its importance in SG7.AF binding. These may be eventually linked to the surface covalently as shown in Figure 10 or remain associated only through properdin binding. Properdin is known to form trimers and tetramers that interact with more than one complement complex simultaneously (37), possibly facilitating oligomerization. However, albicin inhibits erythrocyte lysis effectively in the presence or absence of properdin showing that albicin-inhibited complexes can orient appropriately to form oligomers on cell surfaces in the absence of properdin. Additionally, albicin does not enhance binding of C3b or C3bB to properdin SPR surfaces suggesting that it does not promote oligomerization of C3b before activation of the mature convertase (14). It seems more likely that rather than helping to orient C3b, C3bB, or C3bBb during assembly of the inhibited oligomeric complex, properdin may compensate for the lower affinity of SG7.AF by further stabilizing the inhibited complex after assembly (Fig. 10). Without properdin, C3bBb monomers may have more freedom to move into catalytically active orientations, while properdin binding may slow down this process by making the structure more rigid (Fig. 10).

Although the SG7 salivary protein family is distributed throughout the genus *Anopheles*, potent salivary anticomplement activity is more phylogenetically restricted (28). In the subgenus *Nyssorynchus*, we find that albicin from *An. albimanus* inhibits activation of the AP by binding the C3bBb complex. Extracts from a second *Nyssorynchus* species, *An. aquasalis*, have also been found to contain inhibitors of the AP that are not yet confirmed to be albicin homologs (38). Here we also show that *An. freeborni* (subgenus *Anopheles*) contains SG7.AF, which inhibits the AP in a manner similar to that of albicin and that anophensin from *An. stephensi* is a far weaker inhibitor of the AP than albicin or SG7.AF. The latter observation indicates that a functional divergence within the genus has occurred as is suggested by the activity of anophensin against proteases of the contact pathway of coagulation. SG7 family members are quite similar to one another structurally, but amino acid sequence divergence suggests that specific side chain interactions are key determinants for the observed differences in target affinity. However, the alternative structure of the extended form of anophensin implies that large changes in backbone conformation may allow additional functional diversification in the 30 kDa family.

## Experimental Procedures

### Purified proteins and sera

Human C3, C3b, C3a, factor B, factor D, properdin, NHS, PDS, polyclonal rabbit antihuman C3a, goat antihuman factor B, goat antihuman properdin, goat antihuman factor D, and goat antihuman C3 were obtained from Complement Technologies.

### Expression of recombinant proteins

Optimized synthetic cDNAs encoding albicin, SG7.AF, and anophensin having signal sequences replaced by an ATG initiation codon were inserted into the pET-17b expression vector, which was used to transform BL-21(DE3)pLysS *E. coli*. After culturing and induction, inclusion bodies were prepared by previously established methods (2). After solubilization in 6 M guanidine HCl, 20 mM Tris pH 8.0, the proteins were refolded by dilution into a buffer containing 300 mM arginine, 20 mM Tris-HCl pH 8, 2 mM cystamine pH 8.0. After concentration, the proteins were purified by a combination of gel filtration chromatography on Sephacryl S-100, ion exchange chromatography on Q-Sepharose, and hydrophobic interaction chromatography on Phenyl Sepharose. Following purification, SG7 proteins were stored in 20 mM Tris-HCl, 300 mM NaCl pH 8. Gel filtration on Superdex 75 indicated that the proteins were in the monomeric state (Fig. S5). A variant of anophensin with the N-terminus extended by the retention of three amino acids from the signal peptide was produced in same manner as wild-type anophensin (Fig. S4). A selenomethionine derivative of this protein was produced in the auxotrophic *E. coli* cell line B834(DE3)pLysS using the SelenoMet media system (Molecular Dimensions), refolded, and purified as described above.

### Assay of AP-mediated hemolysis of rabbit erythrocytes.

The hemolysis of rabbit erythrocytes in the presence of human serum was used to evaluate the inhibition of the AP by albicin, SG7.AF, and anophensin. Rabbit erythrocytes (Complement Technologies) were washed three times with five volumes of Mg-EGTA solution (1 mM HEPES, 30 mM NaCl, 10 mM EGTA, 7 mM MgCl_2_, 3% glucose, 0.02% gelatin pH 7.4). After centrifugation (600*g*, 10 min, 4 °C), the cell concentration was adjusted to 1 × 10^8^/ml. Assays were carried out in microcentrifuge tubes containing 25 μl NHS or PDS diluted 1:2 or 1:20 in Mg-EGTA solution, 25 μl of washed rabbit erythrocytes, and 12.5 μl of different concentrations of albicin or SG7.AF diluted in phosphate-buffered saline (PBS). Samples were incubated at 37 °C for 30 min in the presence of NHS or 40 min when PDS was used, followed by addition of 250 μl of ice-cold PBS. Samples were briefly centrifuged (1000 rpm, 3 s, 4 °C), and 200 μl of the supernatant was transferred to an ELISA plate and read at 415 nm. The possibility of inhibition of the CP was also measured by hemolysis assays. In this case, Ab-sensitized sheep erythrocytes (Complement Technologies) were used, and the assays were conducted as described previously (14). Data analysis was carried out as follows: the measurement of negative controls (erythrocytes incubated in the absence of serum and SG7.AF) was subtracted from all data points, and hemolysis inhibition was determined by the ratio of erythrocytes incubated with serum and SG7.AF by the positive control (erythrocytes incubated with serum only). AP experiments were carried out in triplicate using three independent preparations of erythrocytes and CP experiments in duplicate.

### Evaluation of factor B and C3 cleavage in erythrocyte lysis supernatants

AP-mediated hemolysis assays were performed as described above, using 1:20 diluted NHS and 40 nM of SG7.AF. Tubes were incubated at 37 °C for 0, 30, or 60 min, and 5 μl of the supernatant was collected and separated in a 10% NuPAGE gel. Separated proteins were transferred to a nitrocellulose membrane and blocked for 1 h at room temperature with 10% nonfat dry milk in 0.05% tween 20 in PBS (PBS-T). After blocking, the membrane was washed three times with PBS-T and incubated 16 h at room temperature with rabbit antihuman C3a or goat antihuman factor B (1:10,000) diluted in PBS-T containing 1% BSA. After incubation, the membrane was washed with PBS-T and incubated for 1 h at room temperature with antigoat or anti-rabbit IgG diluted 1:7500 in PBS-T. Detection was carried out by the addition of the Super Signal West Pico PLUS substrate (Pierce, Thermo Fisher) and exposed using an iBrightFL1000 (Thermo Fisher). Purified C3a or factor Bb (15 ng) was used as positive control.

### Inhibition of C3bBb activity and factor B activation

C3bBb was assembled by incubating 200 nM human C3b, 100 nM factor B, and 50 nM factor D in 0.01 M HEPES, 0.15 M NaCl pH 7.4 containing 2 mM MgCl_2_ (HBS/MgCl_2_) for 2 min. After addition of 5 mM EDTA, C3 was added at concentrations of 0, 100, and 200 nM along with albicin or SG7.AF at concentrations of 2 μM. The mixture was then incubated at room temperature for 20 min and stopped by adding 5x NuPAGE sample buffer to the reaction mixture. C3 cleavage was evaluated by western blot after SDS-PAGE performed under reducing conditions, using rabbit antihuman C3a for detection.

Inhibition of factor B activation was evaluated by incubating 200 nM C3b, 100 nM factor B, and 50 nM factor D in the presence of 2 μM of SG7.AF or albicin in HBS/MgCl_2_ for 0, 20, or 40 min at 37 °C. The reaction products were detected by western blotting as described above using antihuman factor B as the primary antibody.

### Deposition and displacement of complement components from agarose-coated plates

ELISA plates (Corning) were filled with 100 μl of 0.1% agarose solution and incubated at 37 °C until completely dry. NHS (20%) in HMEBN buffer (5 mM HEPES, 7 mM MgCl_2_, 10 mM EGTA, 5 mg/ml BSA, and 140 mM NaCl, pH 7.4) with different concentrations of SG7.AF was incubated at 37 °C for 30 min, washed three times with PBS-T, and incubated with anti-C3 (1:5000), anti–factor B (1:200) or antiproperdin (1:200) in PBS-T for 1 h at 37 °C. After three more washes with PBS-T, wells were treated with antigoat IgG conjugated with peroxidase (Sigma) diluted 1:5000 in PBS-T for 1 h at 37 °C. Wells were washed three more times, and 100 μl of the pNPP substrate (Sigma) was added and the plate incubated at 37 °C for 30 min. Finally, the plate was read at 405 nm. The displacement assay was carried out in a similar fashion, in which NHS (20%) was incubated in the absence of SG7.AF for 30 min at 37 °C followed by incubation with 0.8 μM SG7.AF for 30 min at 37 °C. Data analysis was carried out as follows: the measurement of negative controls (wells treated with buffer only) was subtracted from all data points, and deposition inhibition was determined by the ratio of wells treated with NHS and SG7.AF by the positive control (well treated with NHS only). All experiments were carried out in duplicate.

### Surface plasmon resonance

The assays were performed on a Biacore T100 instrument (GE Healthcare). Properdin at a concentration 20 μg/ml in 10 mM sodium acetate, pH 5.0 was immobilized on a CM5 sensor chip (GE Healthcare) to a level of 5000 RU using the amine-coupling method. To evaluate the effect of SG7.AF on C3bBb stabilization, purified C3b (20 μg/ml), factor B (20 μg/ml), and factor D (2 μg/ml) in HBS/MgCl_2_ were injected at 30 μl/min for 120 s in the absence or presence of different concentrations of purified recombinant SG7.AF or albicin. Surface regeneration was carried out with 10 mM glycine pH 2.5 for 10 s.

### Analysis of inhibitor-bound C3bBb complexes by gel filtration chromatography

Complexes of C3bBb containing albicin were prepared by mixing C3b, factor B, factor D, and recombinant albicin in an approximate molar ratio of 1:2:1:5 in HBS/MgCl_2_. After 15 min of incubation at room temperature, the mixture was separated by gel filtration chromatography on a Superdex 200 (10/300) column equilibrated with HBS/MgCl_2_. After elution, fractions were analyzed by silver staining of SDS-PAGE gels run under reducing conditions.

### Crystallization of albicin, SG7.AF and anophensin

Albicin, SG7.AF, and anophensin were crystallized using the hanging drop vapor diffusion method at 25 °C. Albicin at a stock concentration of 10 mg/ml was crystallized from 10% to 20% PEG 3350, 0.2 M NH_4_F at pH 7, and SG7.AF was crystallized from 1.8 M (NH_4_)_2_SO_4_ from pH 7 to 9 containing 0.1 M HEPES (pH 7), 0.1 M Tris HCl (pH 8), and 0.1 M Bicine (pH 9). Lengthened anophensin and its selenomethionine derivative, at a concentration of 12 mg/ml, were crystallized from 5% to 7.5% PEG 10,000, 0.1 M Tris pH 8.5. Albicin crystals were prepared for data collection by flash cooling in liquid nitrogen after soaking briefly in a cryopreservative solution of 20% PEG 3350, 0.5 M NH_4_Br, 10% glycerol at pH 7. SG7.AF crystals were prepared for flash cooling after soaking in a cryopreservative of 3.7 M (NH_4_)_2_SO_4_ at pH 9. Anophensin crystals were soaked in a cryopreservative of 20% PEG 10,000, 0.1 M Tris pH 8.5, 15% glycerol.

### Data collection and structure solution

Diffraction data were collected at beamline 22-ID of the Southeast Regional Collaborative Access Team (SER-CAT) at the Advanced Photon Source, Argonne National Laboratory (Table 1). Images were processed using XDS/XSCALE and HKL2000 (39, 40). Albicin was crystallized in the space group P2_1_2_1_2 with three monomers contained in the asymmetric unit. The structure of albicin was solved using single anomalous diffraction (SAD) methods with data collected from NH_4_Br-soaked crystals and processed with Shelx C, D, and E (41). The initial model was built with Buccaneer and completed using cycles of manual rebuilding with Coot and refinement using Phenix with a TLS model applied (42, 43, 44). The quality of the structure was evaluated using MolProbity (45). SG7.AF was crystallized in the space group P4_1_2_1_2 with a single monomer in the asymmetric unit. The structure was determined by molecular replacement with Phaser (46), using the albicin monomer as a search model, and then refined using Phenix as above. Anophensin containing an N-terminal extension of three residues was crystallized in the space group P2_1_2_1_2 with three monomers in the asymmetric unit. The structure of anophensin was determined using SAD methods in Phenix Autosol (42) with diffraction data from the selenomethionine derivative. The structure was built manually in Coot and refined using Phenix with a TLS model applied.

### Data availability

Coordinates and structure factors for albicin, SG7.AF, and anophensin were submitted to the wwPDB with the accession codes 6XKE, 6XL7, and 6XMB, respectively. Any data not contained in the article are available from the authors.

## Conflict of Interest

The authors declare that they have no conflicts of interest with the contents of this article.

## References

[1] Ribeiro J.M., Mans B.J., Arca B. (2010). An insight into the sialome of blood-feeding nematocera. Insect Biochem. Mol. Biol..

[2] Alvarenga P.H., Francischetti I.M., Calvo E., Sa-Nunes A., Ribeiro J.M., Andersen J.F. (2010). The function and three-dimensional structure of a thromboxane A2/cysteinyl leukotriene-binding protein from the saliva of a mosquito vector of the malaria parasite. PLoS Biol..

[3] Champagne D.E., Smartt C.T., Ribeiro J.M., James A.A. (1995). The salivary gland-specific apyrase of the mosquito Aedes aegypti is a member of the 5'-nucleotidase family. Proc. Natl. Acad. Sci. U. S. A..

[4] Francischetti I.M., Valenzuela J.G., Ribeiro J.M. (1999). Anophelin: kinetics and mechanism of thrombin inhibition. Biochemistry.

[5] Ma D., Xu X., An S., Liu H., Yang X., Andersen J.F., Wang Y., Tokumasu F., Ribeiro J.M., Francischetti I.M., Lai R. (2011). A novel family of RGD-containing disintegrins (Tablysin-15) from the salivary gland of the horsefly Tabanus yao targets alphaIIbbeta3 or alphaVbeta3 and inhibits platelet aggregation and angiogenesis. Thromb. Haemost..

[6] Jablonka W., Pham V., Nardone G., Gittis A., Silva-Cardoso L., Atella G.C., Ribeiro J.M., Andersen J.F. (2016). Structure and ligand-binding mechanism of a cysteinyl leukotriene-binding protein from a blood-feeding disease vector. ACS Chem. Biol..

[7] Kotsyfakis M., Sa-Nunes A., Francischetti I.M., Mather T.N., Andersen J.F., Ribeiro J.M. (2006). Antiinflammatory and immunosuppressive activity of sialostatin L, a salivary cystatin from the tick Ixodes scapularis. J. Biol. Chem..

[8] Mans B.J., Calvo E., Ribeiro J.M., Andersen J.F. (2007). The crystal structure of D7r4, a salivary biogenic amine-binding protein from the malaria mosquito Anopheles gambiae. J. Biol. Chem..

[9] Qureshi A.A., Asahina A., Ohnuma M., Tajima M., Granstein R.D., Lerner E.A. (1996). Immunomodulatory properties of maxadilan, the vasodilator peptide from sand fly salivary gland extracts. Am. J. Trop. Med. Hyg..

[10] Couvreur B., Beaufays J., Charon C., Lahaye K., Gensale F., Denis V., Charloteaux B., Decrem Y., Prevot P.P., Brossard M., Vanhamme L., Godfroid E. (2008). Variability and action mechanism of a family of anticomplement proteins in Ixodes ricinus. PLoS One.

[11] Ferreira V.P., Fazito Vale V., Pangburn M.K., Abdeladhim M., Mendes-Sousa A.F., Coutinho-Abreu I.V., Rasouli M., Brandt E.A., Meneses C., Lima K.F., Nascimento Araujo R., Pereira M.H., Kotsyfakis M., Oliveira F., Kamhawi S. (2016). SALO, a novel classical pathway complement inhibitor from saliva of the sand fly Lutzomyia longipalpis. Sci. Rep..

[12] Jore M.M., Johnson S., Sheppard D., Barber N.M., Li Y.I., Nunn M.A., Elmlund H., Lea S.M. (2016). Structural basis for therapeutic inhibition of complement C5. Nat. Struct. Mol. Biol..

[13] Mendes-Sousa A.F., do Vale V.F., Silva N.C.S., Guimaraes-Costa A.B., Pereira M.H., Sant'Anna M.R.V., Oliveira F., Kamhawi S., Ribeiro J.M.C., Andersen J.F., Valenzuela J.G., Araujo R.N. (2017). The sand fly salivary protein lufaxin inhibits the early steps of the alternative pathway of complement by direct binding to the proconvertase C3b-B. Front. Immunol..

[14] Mendes-Sousa A.F., Queiroz D.C., Vale V.F., Ribeiro J.M., Valenzuela J.G., Gontijo N.F., Andersen J.F. (2016). An inhibitor of the alternative pathway of complement in saliva of new world anopheline mosquitoes. J. Immunol..

[15] Reichhardt M.P., Johnson S., Tang T., Morgan T., Tebeka N., Popitsch N., Deme J.C., Jore M.M., Lea S.M. (2020). An inhibitor of complement C5 provides structural insights into activation. Proc. Natl. Acad. Sci. U S A..

[16] Valenzuela J.G., Charlab R., Mather T.N., Ribeiro J.M. (2000). Purification, cloning, and expression of a novel salivary anticomplement protein from the tick, Ixodes scapularis. J. Biol. Chem..

[17] Ricklin D., Hajishengallis G., Yang K., Lambris J.D. (2010). Complement: a key system for immune surveillance and homeostasis. Nat. Immunol..

[18] Kemper C., Atkinson J.P., Hourcade D.E. (2010). Properdin: emerging roles of a pattern-recognition molecule. Annu. Rev. Immunol..

[19] Spitzer D., Mitchell L.M., Atkinson J.P., Hourcade D.E. (2007). Properdin can initiate complement activation by binding specific target surfaces and providing a platform for de novo convertase assembly. J. Immunol..

[20] Hourcade D.E., Akk A.M., Mitchell L.M., Zhou H.F., Hauhart R., Pham C.T. (2016). Anti-complement activity of the Ixodes scapularis salivary protein Salp20. Mol. Immunol..

[21] Tyson K.R., Elkins C., de Silva A.M. (2008). A novel mechanism of complement inhibition unmasked by a tick salivary protein that binds to properdin. J. Immunol..

[22] Park D.H., Connor K.M., Lambris J.D. (2019). The challenges and promise of complement therapeutics for ocular diseases. Front. Immunol..

[23] Woodruff T.M., Nandakumar K.S., Tedesco F. (2011). Inhibiting the C5-C5a receptor axis. Mol. Immunol..

[24] Hourcade D.E. (2006). The role of properdin in the assembly of the alternative pathway C3 convertases of complement. J. Biol. Chem..

[25] Rooijakkers S.H., Wu J., Ruyken M., van Domselaar R., Planken K.L., Tzekou A., Ricklin D., Lambris J.D., Janssen B.J., van Strijp J.A., Gros P. (2009). Structural and functional implications of the alternative complement pathway C3 convertase stabilized by a staphylococcal inhibitor. Nat. Immunol..

[26] Holm L. (2020). DALI and the persistence of protein shape. Protein Sci..

[27] Rooijakkers S.H., Milder F.J., Bardoel B.W., Ruyken M., van Strijp J.A., Gros P. (2007). Staphylococcal complement inhibitor: structure and active sites. J. Immunol..

[28] Arca B., Lombardo F., Struchiner C.J., Ribeiro J.M. (2017). Anopheline salivary protein genes and gene families: an evolutionary overview after the whole genome sequence of sixteen Anopheles species. BMC Genomics.

[29] Isawa H., Orito Y., Iwanaga S., Jingushi N., Morita A., Chinzei Y., Yuda M. (2007). Identification and characterization of a new kallikrein-kinin system inhibitor from the salivary glands of the malaria vector mosquito Anopheles stephensi. Insect Biochem. Mol. Biol..

[30] Calvo E., Tokumasu F., Marinotti O., Villeval J.L., Ribeiro J.M., Francischetti I.M. (2007). Aegyptin, a novel mosquito salivary gland protein, specifically binds to collagen and prevents its interaction with platelet glycoprotein VI, integrin alpha2beta1, and von Willebrand factor. J. Biol. Chem..

[31] Yoshida S., Sudo T., Niimi M., Tao L., Sun B., Kambayashi J., Watanabe H., Luo E., Matsuoka H. (2008). Inhibition of collagen-induced platelet aggregation by anopheline antiplatelet protein, a saliva protein from a malaria vector mosquito. Blood.

[32] Hayashi H., Kyushiki H., Nagano K., Sudo T., Iyori M., Matsuoka H., Yoshida S. (2013). Identification of the active region responsible for the anti-thrombotic activity of anopheline anti-platelet protein from a malaria vector mosquito. Platelets.

[33] Khattab A., Barroso M., Miettinen T., Meri S. (2015). Anopheles midgut epithelium evades human complement activity by capturing factor H from the blood meal. PLoS Negl. Trop. Dis..

[34] el-Lati S.G., Dahinden C.A., Church M.K. (1994). Complement peptides C3a- and C5a-induced mediator release from dissociated human skin mast cells. J. Invest. Dermatol..

[35] Hartmann K., Henz B.M., Kruger-Krasagakes S., Kohl J., Burger R., Guhl S., Haase I., Lippert U., Zuberbier T. (1997). C3a and C5a stimulate chemotaxis of human mast cells. Blood.

[36] Lepow I.H., Willms-Kretschmer K., Patrick R.A., Rosen F.S. (1970). Gross and ultrastructural observations on lesions produced by intradermal injection of human C3a in man. Am. J. Pathol..

[37] van den Bos R.M., Pearce N.M., Granneman J., Brondijk T.H.C., Gros P. (2019). Insights into enhanced complement activation by structures of properdin and its complex with the C-terminal domain of C3b. Front. Immunol..

[38] Mendes-Sousa A.F., Vale V.F., Queiroz D.C., Pereira-Filho A.A., da Silva N.C.S., Koerich L.B., Moreira L.A., Pereira M.H., Sant'Anna M.R., Araujo R.N., Andersen J., Valenzuela J.G., Gontijo N.F. (2018). Inhibition of the complement system by saliva of Anopheles (Nyssorhynchus) aquasalis. Insect Biochem. Mol. Biol..

[39] Kabsch W. (2010). Xds. Acta Crystallogr. D Biol. Crystallogr..

[40] Otwinowski Z., Minor W. (1997). Processing of X-ray diffraction data collected in oscillation mode. Methods Enzymol..

[41] Schneider T.R., Sheldrick G.M. (2002). Substructure solution with SHELXD. Acta Crystallogr. D Biol. Crystallogr..

[42] Adams P.D., Afonine P.V., Bunkoczi G., Chen V.B., Davis I.W., Echols N., Headd J.J., Hung L.W., Kapral G.J., Grosse-Kunstleve R.W., McCoy A.J., Moriarty N.W., Oeffner R., Read R.J., Richardson D.C. (2010). PHENIX: a comprehensive Python-based system for macromolecular structure solution. Acta Crystallogr. D Biol. Crystallogr..

[43] Emsley P., Cowtan K. (2004). Coot: model-building tools for molecular graphics. Acta Crystallogr. D Biol. Crystallogr..

[44] Cowtan K. (2006). The Buccaneer software for automated model building. 1. Tracing protein chains. Acta Crystallogr. D Biol. Crystallogr..

[45] Williams C.J., Headd J.J., Moriarty N.W., Prisant M.G., Videau L.L., Deis L.N., Verma V., Keedy D.A., Hintze B.J., Chen V.B., Jain S., Lewis S.M., Arendall W.B., Snoeyink J., Adams P.D. (2018). MolProbity: more and better reference data for improved all-atom structure validation. Protein Sci..

[46] McCoy A.J., Grosse-Kunstleve R.W., Adams P.D., Winn M.D., Storoni L.C., Read R.J. (2007). Phaser crystallographic software. J. Appl. Crystallogr..

