# Supplementary material for: Salivary complement inhibitors from mosquitoes: Structure and mechanism of action
Source: J Biol Chem. 2020 Nov 24;296:100083. doi: 10.1074/jbc.RA120.015230 (PMC7948415; doi:10.1074/jbc.RA120.015230)
Supplement: Figures S1–S5 [file mmc1.pdf]

**SUPPLEMENTAL INFORMATION**

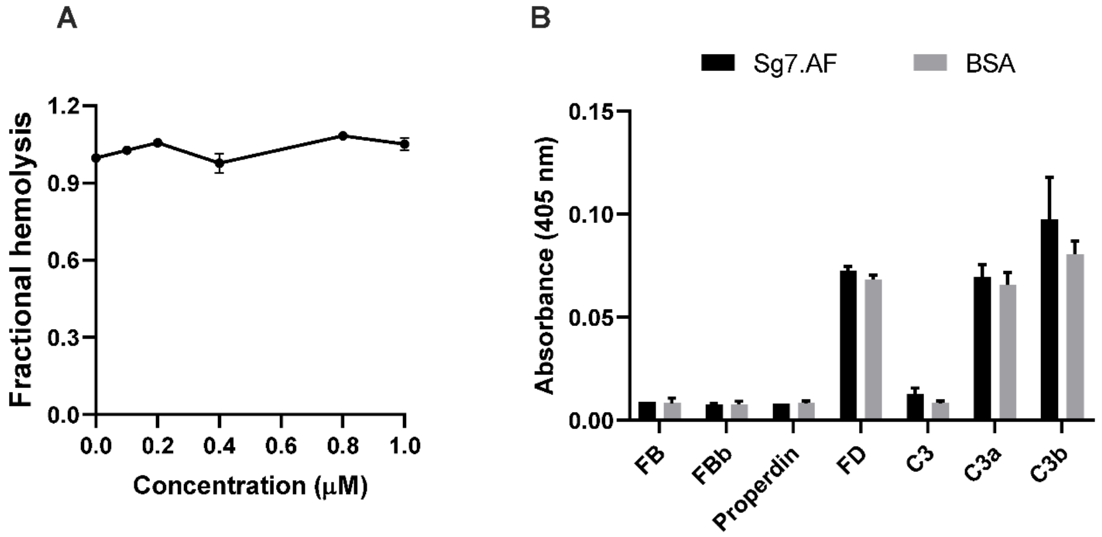


**Fig. S1.** Anticomplement properties of SG7.AF. **(A)** Measurement of CP inhibition by SG7.AF. CP-mediated hemolysis was carried out using Ab-sensitized sheep erythrocytes (1x$10^8$ cell/ml) in $GVB^{++}$ buffer with NHS as indicated in the methods. Graphs show the mean and SEM of two independent experiments. **(B)** Direct binding of complement components to SG7.AF. ELISA plates were coated with 0.5 μg of SG7.AF or BSA overnight at 4ºC and blocked for 2 h at room temperature with 0.05% tween 20, 1% BSA in PBS. After 3 washes with 0.05% tween 20 in PBS samples were incubated with 0.2 μg of factor B (FB), factor Bb (FBb), properdin, factor D (FD), C3, C3a or C3b for 30 min at 37ºC. After 3 washes samples were incubated with primary anti-bodies anti-factor B (1:12000), anti-C3 (1:150000), anti-properdin (1:4000), anti-factor D (1:500) or anti-factor C3a (1:2000) in 0.05% tween 20 in PBS for 30 min at 37ºC. After 3 more washes the samples were incubated with anti-goat or anti-rabbit alkaline phosphatase-conjugated secondary antibody (1:7000) in 0.05% tween 20 in PBS for 30 min at 37ºC. Finally, after 3 more washes 100 μL of the pnPP substrate (Sigma) was added. The plate was incubated for 30 min at 37ºC and read at A405 nm.

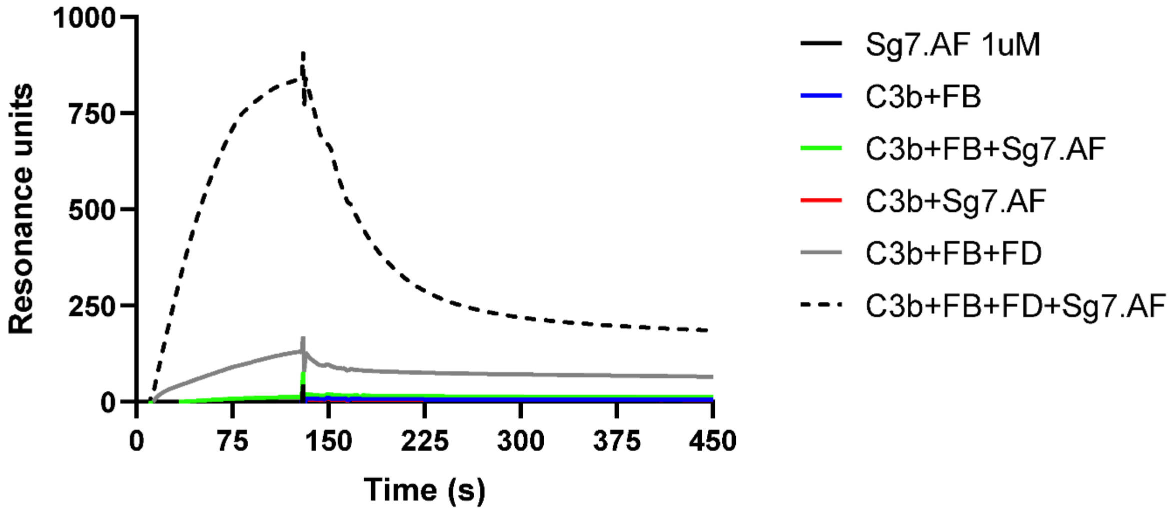


**Fig. S2**. Interaction of C3bBb components and the inhibitor SG7.AF with immobilized properdin as measured with SPR. Concentrations of C3b, factor B (FB), factor D (FD) are the same as in Fig. 5. The concentration of SG7.AF is 1 μM.

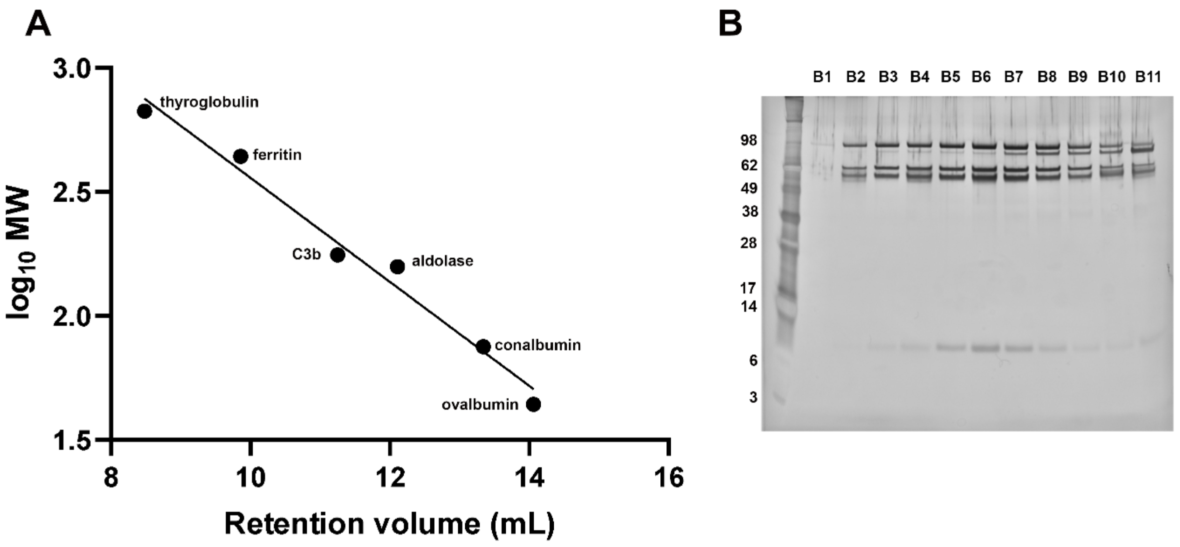


**Fig. S3.** Gel filtration studies of the albicin-bound C3bBb complex. **(A)** Molecular weights of standard proteins vs. retention volume for the Superdex 200 column run under the conditions of Fig. 6. **(B)** Silver stained SDS-PAGE gel shown in Fig. 6 with molecular weight standards (SeeBlue plus 2, Thermo Fisher) shown in the left column.

**A**

```
                    α1                            α2                    α3
albi ANNHIRTVLK LFRTIDLDDS KKSFYLTAAK YGIQTQLREP IIRIVGGYLP STKLSEACVK
free ARKHVQELLK TFRRIDFDET RKSVYLQSAK FGVQSQLREP LTKKVLNYWD DVKLSKTCLD
anop TRKHVQQLMK VFRAIDFDFT KKAFYLHRAK YGVQNQLRNP LYLKAMSLPR SAKLSQPCLN

                 α3                            α4
albi NMISEVYEIE GDFYSKFSYA CEDHAPYSVE CLEDARDDYL TQLVELFKET KKCLRE....
free RMVTKVNDVK ETFYAGFSYA CESHNQYSVD CLEAAKPSYL TALGEIRGET EKCLTTRLK.
anop KMIDEVNDLE STFYAGFSFN CHDHDQYSMD CLEAAEPTYL DGLKKLAAST EQCLVQK...
```

**B**

```
aegyptin RPMPEDEEPV AEGGDDDASG ESEGEEETTD DAGGDGGEEE NEGEEHAGDK DAGGEDTGKE
    aapp RPSDETTDQE SSTELSEDTS DSYHQEEDTS ETGADAGTED GNSEDDSSEL ESSSEE...G
    albi .......... .......... .......... .......... .......... ..........
    free .......... .......... .......... .......... .......... ..........
    anop .......... .......... .......... .......... .......... ..........

aegyptin ENTGHDDAGE EDAGEEDAGE EDAGEEDAGE EDAEKEEGEK EDAGDDAGSD DGEEDSTGGD
    aapp HEDGSEDATG EEGGAGEKGE AG.EEDEAGE EGEAGEEGEA GEEGGAGEEG GAGEEGGADE
    albi .......... .......... .......... .......... .......... ..........
    free .......... .......... .......... .......... .......... ..........
    anop .......... .......... .......... .......... .......... ..........

                                     α1                           α2
aegyptin EGEDNAEDSK GSEKNDPADT YRQVVALLDK DTKVDHIQSE YLRSALNNDL QSEVRVPVVE
    aapp EGSAGEEGGA EGGEESPVNT YHQVHNLLKN IMNVG.TKNN YLKSFILARL QERLMNPTID
    albi .......... .......ANN HIRTVLKLFR TIDLDDSKKS FYLTAAKYGI QTQLREPIIR
    free .......... .......ARK HVQELLKTFR RIDFDETRKS VYLQSAKFGV QSQLREPLTK
    anop .......... ....TEATRK HVQQLMKVFR AIDFDFTKKA FYLHRAKYGV QNQLRNPLYL

            α2                 α3                                α4
aegyptin AIGRIGDYSK IQG.CFKSMG KDVKKVISEE EKKFK.SCMS KKKSEYQCSE DSFAAAKSKL
    aapp LVGSISKYSK IKE.CFDSLA DDVKSLVEKS ETSYE.ECS. KDKNNPHCGS EGTRELDEGL
    albi IVGGYLPSTK LSEACVKNMI SEVYEIEGDF YSKFSYACED HAPYSVECLE DARDDYLTQL
    free KVLNYWDDVK LSKTCLDRMV TKVNDVKETF YAGFSYACES HNQYSVDCLE AAKPSYLTAL
    anop KAMSLPRSAK LSQPCLNKMI DEVNDLESTF YAGFSFNCHD HDQYSMDCLE AAEPTYLDGL

              α4
aegyptin SPITSKIKSC VSSKGR..
    aapp IEREQKLSDC IVEKRDSE
    albi VELFKETKKC LRE.....
    free GEIRGETEKC LTTRLK..
    anop KKLAASTEQC LVQK....
```

**Fig. S4.** Amino acid alignments of SG7 proteins from different *Anopheles* species. **(A)** Alignment of the anticomplement SG7 proteins albicin from *An. albimanus* (albi), SG7.AF from *An. freeborni* (free) and anophensin from *An. stephensi* (anop). **(B)** Alignment of the platelet inhibitory proteins aegyptin from *Ae. aegypti* and AAPP from *An. stephensi* with the SG7 proteins from panel **A**. The sequence of mature anophensin is shown in bold type while the extension of three amino acids at the N-terminal end of

anophensin used in crystallization experiments is not bolded. In both alignments the conserved cysteines are highlighted in black. The boundaries of the α-helices α1-α4 are indicated by solid black lines.

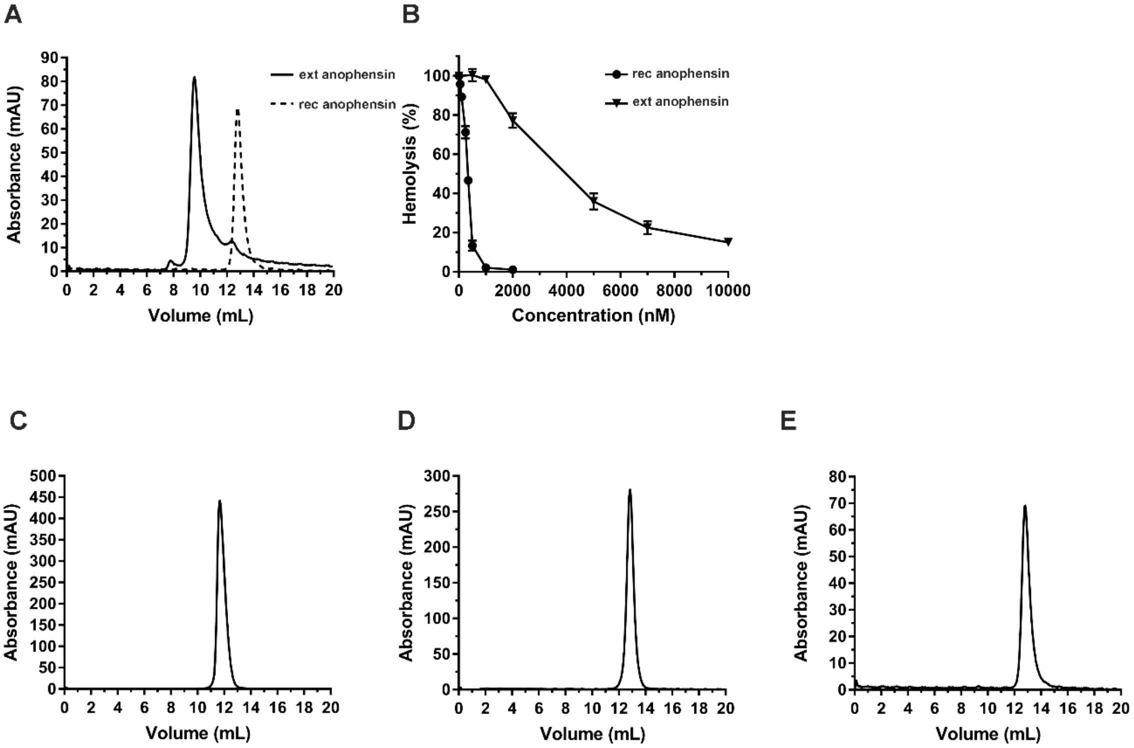


**Fig. S5.** Properties of recombinant SG7 proteins from various species. **(A)** Retention of recombinant anophensin (rec anophensin, dashed line) and extended anophensin (ex anophensin, solid line) on Superdex 75. **(B)** Inhibition of the AP by extended anophensin (inverted triangles) and recombinant anophensin (circles) as indicated by lysis of rabbit erythrocytes. **(C-E)** Gel filtration on Superdex 75 of recombinant albicin **(C)**, SG7.AF **(D)** and anophensin **(E)**. In panels **A** and **C-E** the elution buffer was 20 mM Tris HCl pH 8, 150 mM NaCl.
